# Effect of Donor Age on Endocrine Function of and Immune Response to Ovarian Grafts

**DOI:** 10.3390/ijms25063431

**Published:** 2024-03-19

**Authors:** Monica A. Wall, Mayara Garcia de Mattos Barbosa, Natalie Hanby, Michelle M. Cai, Margaret Brunette, Despina I. Pavlidis, Paula Arrowsmith, Ansen Q. Tan, Marilia Cascalho, Ariella Shikanov

**Affiliations:** 1 Department of Biomedical Engineering, University of Michigan, Ann Arbor, MI 48109, USA; mawall@umich.edu (M.A.W.); nhanby@umich.edu (N.H.); mmcai@umich.edu (M.M.C.); mabrunet@umich.edu (M.B.); dpavlidi@umich.edu (D.I.P.); ansent@umich.edu (A.Q.T.); 2 Department of Microbiology and Immunology, University of Michigan, Ann Arbor, MI 48109, USA; mgarciad@med.umich.edu (M.G.d.M.B.); marilia@med.umich.edu (M.C.); 3 School of Medicine, Yale University, New Haven, CT 06510, USA; 4 Department of Pathology, University of Michigan, Ann Arbor, MI 48109, USA; sdarrow@med.umich.edu; 5 Department of Biomedical Engineering, Northwestern University, Evanston, IL 60208, USA; 6 Department of Surgery, University of Michigan, Ann Arbor, MI 48109, USA; 7 Department of Obstetrics and Gynecology, Cellular & Molecular Biology Program, Department of Macromolecular Science & Engineering, University of Michigan, Ann Arbor, MI 48109, USA

**Keywords:** primary ovarian insufficiency, ovarian transplant, donor age

## Abstract

Premature loss of ovarian function (POI) is associated with numerous negative side effects, including vasomotor symptoms, sleep and mood disturbances, disrupted urinary function, and increased risks for osteoporosis and heart disease. Hormone replacement therapy (HRT), the standard of care for POI, delivers only a subset of ovarian hormones and fails to mimic the monthly cyclicity and daily pulsatility characteristic of healthy ovarian tissue in reproductive-aged individuals whose ovarian tissue contains thousands of ovarian follicles. Ovarian tissue allografts have the potential to serve as an alternative, cell-based HRT, capable of producing the full panel of ovarian hormones at physiologically relevant doses and intervals. However, the risks associated with systemic immune suppression (IS) required to prevent allograft rejection outweigh the potential benefits of comprehensive and dynamic hormone therapy. This work investigates whether the age of ovarian tissue donor animals affects the function of, and immune response to, subcutaneous ovarian grafts. We performed syngeneic and semi-allogeneic ovarian transplants using tissue from mice aged 6–8 (D7) or 20–22 (D21) days and evaluated ovarian endocrine function and immune response in a mouse model of POI. Our results revealed that tissue derived from D7 donors, containing an ample and homogeneous primordial follicle reserve, was more effective in fully restoring hypothalamic–pituitary–ovarian feedback. In contrast, tissue derived from D21 donors elicited anti-donor antibodies with higher avidity compared to tissue from younger donors, suggesting that greater immunogenicity may be a trade-off of using mature donors. This work contributes to our understanding of the criteria donor tissue must meet to effectively function as a cell-based HRT and explores the importance of donor age as a factor in ovarian allograft rejection.

## 1. Introduction

Ovarian tissue cryopreservation and auto-transplantation (OTCT) has recently been designated as an approved treatment for pediatric cancer patients at risk of primary ovarian insufficiency (POI) [1,2]. OTCT is a state-of-the-art technique that offers patients both preservation of biological fertility and restoration of physiologic ovarian endocrine function.

Restoration of ovarian hormones is essential for ensuring quality of life in POI patients due to the crucial role ovarian hormones play in maintaining homeostasis throughout the body. Premature loss of ovarian endocrine function is associated with numerous negative side effects—which present primarily in non-reproductive organ systems—including vasomotor symptoms, sleep and mood disturbances, disruptions to urinary function, and increased risks for osteoporosis and heart disease [3]. OTCT is a promising approach for addressing this diverse set of side effects, given the ability of transplanted ovarian tissue to produce the full panel of ovarian hormones at physiologic levels and intervals, thereby restoring the reciprocity of the hypothalamus–pituitary–ovary (HPO) axis. In comparison, hormone replacement therapy (HRT), the only clinically approved treatment for POI, delivers only estradiol (and progesterone when indicated) [4,5]. This course of treatment does not recapitulate the daily pulsatility and monthly cyclicity characteristics of ovarian hormone production.

Unfortunately, OTCT is only available to a fraction of POI patients—those with the opportunity and means to undergo surgical retrieval and cryopreservation of their own ovarian tissue before beginning gonadotoxic treatments. To address this gap, transplantation of donor-derived, ovarian tissue has been proposed and tested in several mammalian species [6,7,8,9], including humans [10,11]. Yet, this approach remains experimental, and is therefore inaccessible to many patients, primarily due to the severe side effects associated with immunosuppression from the drugs [12] with which allogeneic-organ recipients are typically treated indefinitely to protect the allograft from being damaged by their own immune system. Over the last decade, significant attention has been dedicated to uncovering methods for immunosuppressive weaning after heart, liver, and kidney recipients enter the maintenance phase of their immunosuppressive regimens [13]. Reliable protocols for immunosuppressive weaning and/or biomarkers for identifying patients likely to respond positively to decreased immunosuppression would be a tremendous step towards expanding the patient population eligible to receive ovarian allotransplants for whom this is not yet a reality. Despite this, ovarian transplantation research in recent years has focused primarily on auto-transplantation, and a significant gap still exists in our understanding of the factors that may influence the success or failure of ovarian allografts. With respect to graft function, there is a general consensus that transplantation of ovarian tissue possessing a greater number of primordial follicles (i.e., tissue derived from younger, reproductive-age individuals) is correlated with superior restoration of endocrine function (often measured by the return of menses), graft longevity, and pregnancy rates [14,15]. At present, it is, however, unclear how the number and maturity of follicles present at the time of transplant influence the endocrine function conferred by the graft. This is an important clinical consideration for determining donor eligibility. Follicles at various stages exist simultaneously within the ovary and follicle density and distribution, the major determinants of graft success or failure, vary widely between individuals. However, to our knowledge no studies have investigated potential links between follicle maturity or number and the immune response to ovarian grafts. Furthermore, it is well-established that angiogenesis and immune cell recruitment increase considerably in the later stages of folliculogenesis [16,17,18], suggesting that the developmental stages of the follicles present within ovarian tissue may dictate the type and intensity of the alloimmune response mounted. For these reasons, investigating the function of, and immune response to, ovarian allografts containing differing combinations of immature and mature follicles is necessary for establishing ovarian tissue donor criteria, and may also uncover organ-specific factors governing the feasibility of immunosuppressive weaning following ovarian allotransplantation.

We used a mouse model to study the effects of follicle maturity and density on graft function and immunogenicity. In mice, complete folliculogenesis from the primordial stage to ovulation takes approximately 21 days, during which follicles increase in size, secrete hormones, and eventually rupture, releasing an oocyte. Ovaries from 6–8-day-old (D7) pups are small and contain a homogeneous population of predominantly primordial follicles. In contrast, ovaries from 20–22-day-old (D21) mice are larger and contain follicles at all stages of development. We asked whether transplantation of immature ovary tissue (D7) was less or more efficacious than transplantation of mature ovary tissue (D21) in the re-establishment of ovarian function in oophorectomized mice. We also asked whether immature ovary tissue (D7) or mature ovary tissue (D21) evoked allospecific immunity limiting graft life. We hypothesized that transplantation of ovaries from older, D21, donors would restore endocrine function faster but also accelerate rejection due to the presence of hormone-producing, late-stage follicles in D21 tissue compared to ovaries from younger D7 donors that contain only immature, paracrine-controlled ovarian follicles. To test these hypotheses, we transplanted ovaries from D7 and D21 pups into ovariectomized adult hosts and compared the restoration of ovarian endocrine function as well as investigating immunity to the grafts. 

## 2. Results and Discussion

**Figure 1:**
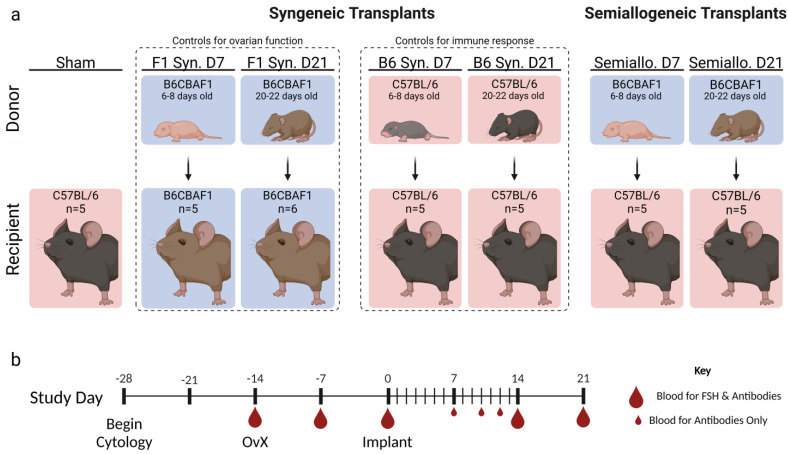
Experimental Design. (**a**) Ovarian tissue from mice, either 6–8 (D7 groups) or 20–22 days old (D21 groups), was transplanted subcutaneously into ovariectomized adult mice. To ensure the volume of transplanted tissue was equal across all subjects, D7 groups received two whole ovaries and D21 groups received two quarter ovaries. Syngeneic transplants were performed with both C57BL/6 (B6) mice (serving as controls for host immune response) and B6CBAF1 (F1) mice (serving as controls for ovarian endocrine function). For semi-allogeneic transplants, B6 hosts received ovarian tissue from F1 pups. Vaginal cytology was collected from 2 weeks before ovariectomy to 21 days after transplant. (**b**) Blood was collected at specified time points for analysis of serum follicle-stimulating hormone (FSH) and/or donor-specific Immunoglobulin G (IgG).

Ovarian tissue was isolated from mice that were 6–8 (D7) or 20-22 (D21) days old and transplanted subcutaneously into genetically identical (syngeneic) or mismatched (semi-allogeneic) ovariectomized-adult mice (Figure 1a). Ovaries from D7 mice, measuring approximately 0.16 mm^3^, are significantly smaller than ovaries from D21 mice, which measure approximately 3.11 mm^3^. Given that rejection kinetics are dose dependent [19], the volume of tissue transplanted (rather than total number of ovaries) was standardized across groups to ensure that any differences observed in rejection kinetics could be attributed to donor-age-related differences rather than graft size. To achieve a standard graft size across all groups, recipients in the “D7 group” were implanted with two whole D7 ovaries, while D21 ovaries were first cut into quarters and 2 pieces, each approximately ¼ of an ovary, were implanted in the “D21 group” recipients (Figure S1). Dissection of D21 ovaries in this manner also mimics dissection of the ovarian cortex that is performed clinically prior to cryopreserving human ovarian tissue.

### 2.1. Restoration of Estrous Cyclicity and HPG Axis by Syngeneic and Allogeneic Grafts

**Figure 2:**
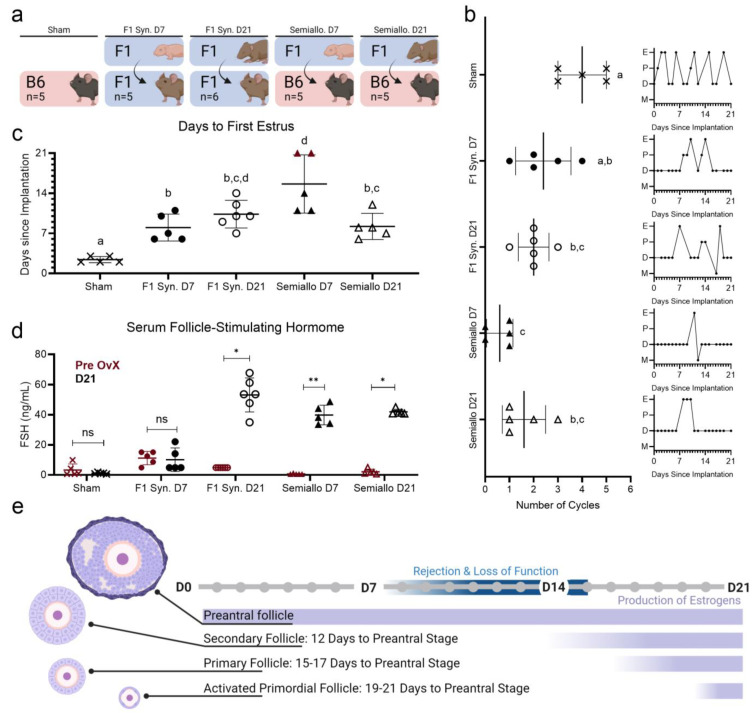
Restoration of endocrine function after ovarian transplantation in syngeneic (Syn) and semiallogeneic (semiallo) hosts (**a**) Schematic of groups for which ovarian function was compared. (**b**) Number of cycles and representative traces of estrus cycles following ovarian transplant (E = estrus, M = metestrus, D = diestrus, and P = proestrus). Rejection of ovarian tissue in semi-allogeneic groups resulted in fewer estrus cycles observed in these hosts. Bars indicate mean ± SD. Statistical significance was determined using the Kruskal–Wallace test and Dunn’s correction for multiple comparisons with *p* < 0.05, letters represent statistically significant differences, where groups that share a letter are not sta-tistically significantly different and groups that do not share a letter are statistically significantly different at a *p* value of at least 0.05 (**c**) Days elapsed between ovarian transplant and resumption of estrous cyclicity. Bars indicate median ± IQR and red points indicate mice that never entered estrus (censored subjects). Log-rank tests were performed on the Kaplan Meier Survival curves of each group with *p* < 0.05 used to determine statistical significance. (**d**) Serum FSH before ovariectomy (red) and 21 days after ovarian transplant (black). Only D7 syngeneic transplants were capable of suppressing FSH secretion to pre-OvX levels. Bars indicate mean ± SD. Statistical significance was determined using the Kruskal–Wallace test with ns representing *p* < 0.05, * representing 0.01 < *p* < 0.05, and ** representing 0.001 < *p* < 0.01. (**e**) Comparison of time required for follicles at different developmental stages to begin producing estrogens with time required for rejection or impaired graft function. D21 ovaries contain preantral follicles and are capable of secreting estrogens at the time of transplant. D7 ovaries contain only immature follicles and may not reach sufficient maturity to induce cyclicity before immune rejection and loss of tissue function.

The restoration of endocrine function in our mouse model of POI was evaluated using two metrics: (1) estrous cyclicity, a measure of ovarian hormone action on the vaginal epithelium; and (2) serum follicle-stimulating hormone, a measure of ovarian hormone feedback to the hypothalamus and pituitary. Mice receiving syngeneic ovarian transplants first entered estrus approximately 9 days after transplant (Figure 2c). Estrous cyclicity was not restored earlier in D21 recipient mice compared to D7 recipient mice despite the fact that D21 ovaries contain mature, hormone-producing follicles at the time of transplant while D7 ovaries contain only immature follicles that must undergo several days of growth before beginning to secrete hormones. We therefore concluded that approximately nine days are required to establish vascularization and exchange between grafted ovarian tissue and the host. In contrast, following semi-allogeneic transplants estrus occurs earlier when tissue is derived from older donors. This again corroborates our hypothesis that vascularization is central to allograft rejection. D21 ovaries contain mature follicles capable of producing hormones at the time of transplant. However, we still see a delay in the resumption of cyclicity in this group, presumably because revascularization is required to transport secreted hormones from the ovarian graft. Additionally, D7 ovaries contain only immature follicles that must undergo additional development before beginning to secrete hormones. This explains why no ovarian function was observed in two mice following transplantation of D7 ovaries. Revascularization occurred before the immature follicles in D7 ovaries began producing hormones. Therefore, grafts were destroyed by infiltrating T cells when the implanted follicles were still in an immature state and rejection occurred before the grafts could engage in hormone production.

We found immunological compatibility between donor and recipient to be a key factor in ensuring the improvement of POI symptoms such as acyclicity and elevated FSH in our model. Once restored, estrous cyclicity persisted in mice who received syngeneic grafts but not in those who received semi-allogeneic grafts. In turn, syngeneic hosts experienced more cycles following transplant (av 2–2.4 cycles) compared to semi-allogeneic hosts (av 0.6–1.6 cycles) (Figure 2b). High terminal serum FSH levels (>40 ng/mL) in semi-allogeneic hosts further confirmed the failure of semi-allogeneic grafts to restore ovarian endocrine function in our model (Figure 2d). In contrast, serum FSH in hosts who received syngeneic D7 grafts significantly decreased (av 10.1 ng/mL) following ovarian transplant, indicating the restoration of reciprocal feedback within the HPG axis.

Notably, terminal FSH levels in syngeneic hosts revealed that donor age does indeed influence the restoration of ovarian function. Serum FSH decreased to physiologically typical levels only in mice with D7 syngeneic transplants. FSH in mice who received D21 syngeneic implants remained elevated despite consistent estrous cyclicity being observed in both D7 and D21 groups. To further confirm that donor age (as opposed to implant duration or tissue volume) was the predominant factor in ensuring FSH suppression, we monitored two additional cohorts of mice for 5 weeks following ovarian transplantation. One cohort received two quarter pieces of D21 ovaries (matching the previously reported D21 group) and the other received eight quarter pieces of D21 ovaries (to match the two ovaries transplanted to the initial D7 group). Even with increased implant duration and/or tissue volume, ovarian tissue derived from D21 donors was unable to reliably restore negative feedback to the HPG at the same rate observed after the transplantation of D7 ovaries (Figure S2).

We therefore concluded that a sufficiently large pool of immature follicles (which are more frequent in prepubertal ovaries than in post-pubertal ovaries per equal tissue volume) is key in ensuring that hormones are secreted in sufficient quantities to restore negative feedback to the HPG axis.

### 2.2. Revascularization and Integration with the Host for Syngeneic and Allogeneic Grafts

**Figure 3:**
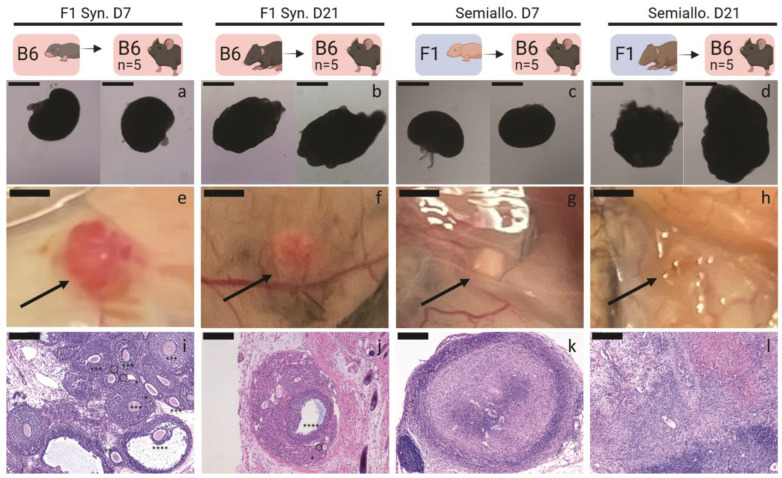
Graft Morphology and Histology for syngeneic (Syn) and semiallogeneic (semiallo) transplants. (**a**–**d**) Isolated ovaries prior to implantation. Ovarian tissue from mice 6–8 (D7)- or 20–22 (D21)-days old was transplanted subcutaneously into ovariectomized adult hosts. To match the volume of transplanted tissue across all groups, D7 groups received two whole D7 ovaries (**a**,**c**) and D21 groups received two quarter-D21 ovaries (**b**,**d**); scale bars = 500 μm. (**e**–h) Ovarian grafts after 21 days in vivo. Semi-allogeneic grafts (**g**,**h**) appear white and shrunken while syngeneic grafts (**e**,**f**) are pink and well-vascularized scale bars = 1 mm. (**i**–**l**) Hematoxylin and eosin staining of explanted ovarian grafts. Semi-allogeneic grafts (**k**,**l**) contain no healthy follicles, but showed dense nuclear staining consistent with immune cell infiltration and graft rejection. In contrast, syngeneic grafts (**i**,**j**) contain healthy follicles at various stages of development (Primordial follicle (dashed circle), primary follicle (*), secondary follicle (**), preantral follicle (***), antral follicle (****)) scale bars = 200 μm.

We analyzed revascularization of the implanted ovarian tissue by histological analysis of the retrieved grafts. Syngeneic D7 (Figure 3e) and D21 (Figure 3f) ovarian tissue retrieved 21 days after transplantation appeared pink, indicating healthy, revascularized tissue. Healthy follicles at various stages of development were present in histological images (Figure 3i,j). Conversely, semi-allogeneic D7 (Figure 3g) and D21 (Figure 3h) ovarian tissue appeared white and shrunken, suggesting limited revascularization and also tissue necrosis. Consistent with the transient ovarian function observed in mice who received semi-allografts, we did not identify healthy follicles in histological sections of semi-allogeneic grafts (Figure 3k,l).

### 2.3. Allogeneic Grafts Induce an Immune Reaction

**Figure 4:**
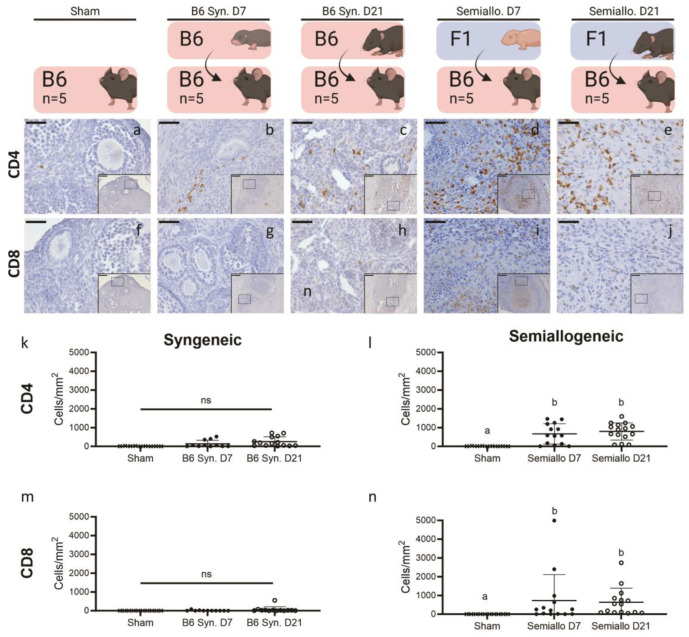
Presence of CD4+ and CD8+ cells at the graft site for syngeneic (Syn) and semiallogeneic (semiallo) transplants. (**a**–**j**) Immunohistochemical staining of explanted ovarian grafts. Brown staining indicates cells positive for CD4 (**a**–**e**) or CD8 (**f**–**j**); scale bars = 50 μm, insets = 200 μm. (**k**–**n**) Density of CD4 and CD8 positive cells at the graft site. Semi-allogeneic grafts had significantly increased densities of CD4+ (**i**) and CD8+ cells. (**n**) T cell comparison with native ovaries. This increased T cell density is consistent with cell-mediated rejection at the site of transplant. In contrast, neither CD4+ (**k**) nor CD8+ (**m**) T cell densities were significantly elevated in syngeneic grafts compared to the native ovary. Bars indicate mean ± SD. Statistical significance was determined using the Kruskal–Wallace test and Dunn’s correction for multiple comparisons with *p* < 0.05 considered significant, ns represents *p* > 0.05, letters represent statistically significant differences, where groups that share a letter are not statistically significantly different and groups that do not share a letter are statis-tically significantly different at a *p* value of at least 0.05.

We investigated anti-graft cellular immunity by counting CD4+ and CD8+ T cells, detected by IHC, in three representative slides per mouse. Throughout semi-allografts, both CD4+ (Figure 4d,e) and CD8+ (Figure 4i,j) T cells were detected, and their numbers were increased in comparison to native ovaries isolated from intact mice (Figure 4l,n). Elevated T cell numbers within a tissue is a classic hallmark of cell-mediated rejection and their presence in the allografts confirms the presence of graft-specific immune responses. In syngeneic grafts, small areas of CD4+ T cells were observed scattered throughout grafts (Figure 4b,c,k), but very few, if any, CD8+ T cells were present (Figure 4g,h,m). The same was true of the native ovary, with few CD4+ T cells found scattered throughout the ovarian stroma (Figure 4a) but virtually no CD8+ T cells found (Figure 4f).

**Figure 5:**
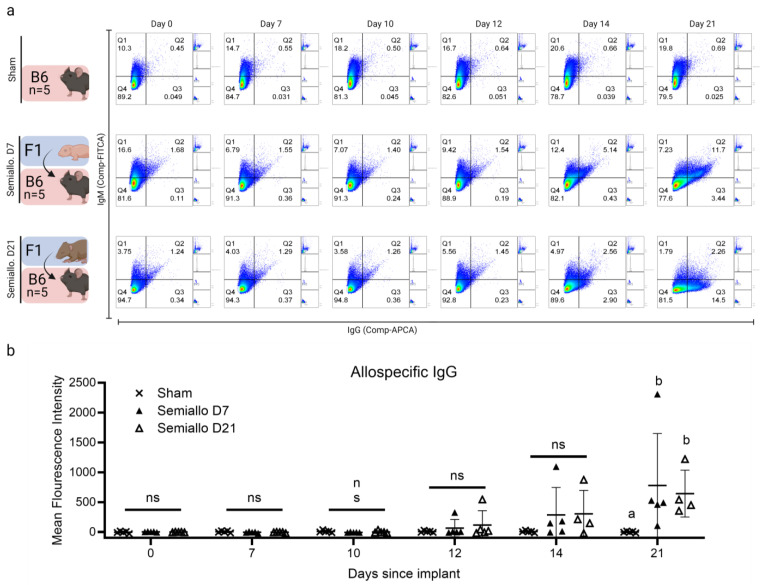
Production of allospecific antibodies after ovarian transplantation to semiallogeneic (semiallo) hosts. Flow cytometry of donor thymocytes incubated with host serum was used to evaluate the production of donor-specific Immunoglobulin M (IgM) and Immunoglobulin G (IgG) after ovarian transplant. (**a**) Representative scatterplots for each group demonstrate an increase in allospecific antibodies in host serum over the 21 days following ovarian allotransplants; x-axis = (IgG), y-axis = (IgM). (**b**) Presence of IgG in host serum as measured by mean fluorescence intensity (MFI). Allospecific IgG was detectable in the serum of a subset of semi-allogeneic (semiallo) hosts as early as 12 days post-transplantation. Overall, 80% of D7 and 100% of D21 subjects who received semi-allogeneic implants mounted a detectable allospecific antibody by 21 days post-transplantation. The fluorescence intensity for each subject is normalized to the value recorded at D0. Bars indicate mean ± SD. Statistical significance was determined using the Kruskal–Wallace test and Dunn’s correction for multiple comparisons with *p* < 0.05 considered significant, ns represents *p* > 0.05, letters represent statistically significant differences, where groups that share a letter are not statistically significantly different and groups that do not share a letter are statis-tically significantly different at a *p* value of at least 0.05.

To investigate the time course of the allospecific immune response to D7 and D21 allogeneic ovaries, we measured allospecific antibodies in the serum of recipient mice 7, 10, 12, 14, and 21 days after transplantation. Representative scatterplots for each group (Figure 5a) display the mean fluorescence intensity values corresponding to Immunoglobulin G (IgG) and M (IgM) bound to allogeneic thymocytes on the x and y axes, respectively. Our data demonstrate that semi-allogeneic subcutaneous ovarian transplants consistently trigger an allospecific humoral immune response. However, the temporal kinetics of this response varied greatly between individual recipients. Donor-specific IgG was first detected in mice implanted with semi-allogeneic ovaries 12 days post-transplant. The time between transplant and initial allo-IgG detection was not influenced by donor age as IgG was first detected 12 days after transplant in both the D7 and D21 groups. Despite the variable onset of a detectable allospecific antibody response between individual mice, those transplanted with allogeneic ovarian tissue did consistently develop a graft-specific immune response. By 21 days post-transplantation, 90% of the tested host serum samples had donor-specific IgG (values for one mouse were unavailable due to technical error). The elevated levels of allospecific IgG in mice who received semi-allogeneic transplants in comparison to sham mice (Figure 5b) argues in favor of a specific, anti-graft-allo-immune response. Further investigation indicated that allogeneic IgG develops faster after the implantation of mature (D21) versus immature (D7) ovaries. This was demonstrated in our flow cytometry data by the vast majority (63%) of events in the D21 semi-allogeneic group testing positive for *IgG only* (evident in Figure 5a as skewing of heatmaps into Q3, 4/4 mice) at 21 days post-transplantation. In contrast, the majority (72%) of events in the D7 semi-allogeneic group were positive for *both IgM and IgG* 21 days after transplantation (seen as skewing of heat maps into Q2). The high proportion of events positive for IgG alone in the D21 allogeneic group suggests that the IgG produced in response to D21 ovarian grafts has high avidity for donor-specific antigens. However, tissue grafts are rejected by T cells and not by antibodies. While our data show immunity following D21 ovarian transplant to be more severe, D7 ovaries are still swiftly rejected (even precluding resumption of estrus cyclicity in several mice). We therefore hypothesize that rejection following transplantation depends primarily on the time required for revascularization of the grafted tissue (which provides a pathway for recipient T cells to enter the graft). This hypothesis is supported by the slightly superior estrous cyclicity seen in D21 mice compared to D7. D21 ovaries have larger follicles that secrete hormones immediately upon implantation. As a result, we see transient cyclicality, followed by rejection and the associated revascularization. D7 ovaries only contain immature follicles that require several additional days of development to catch up and reach the same size that D21 ovaries had attained on day 0. Thus, while revascularization and rejection occur on comparatively the same timescales in both groups, follicles in D7 ovaries are much less likely to reach sizes large enough to maintain endocrine function before being rejected.

Reliable protocols for the transplantation of allogeneic ovarian tissue would be a tremendous advancement for individuals who rely on exogenous ovarian hormones. However, an inability to protect grafts from rejection without the use of systemic immunosuppressants means that only patients who have previously cryopreserved their own tissue have access to this alternative HRT treatment. Widespread clinical implementation of ovarian tissue allografting depends on reliable methods to select suitable ovarian tissue donors and to protect tissue from rejection following transplantation.

Ongoing work in our group is focused on preventing ovarian allograft rejection via encapsulation of ovarian tissue in an immuno-isolating hydrogel capsule [8]. We have previously demonstrated the ability of our device to prevent rejection of encapsulated tissue and restore ovarian endocrine function in a mouse model of POI. However, prior to this work our models relied on tissue from prepubertal mice. In this work, we investigate the capacity of tissue from both prepubertal and pubertal donors to restore ovarian endocrine function, an important consideration for clinical translation as human ovarian tissue donors are likely to be of reproductive age. Furthermore, we had not examined the kinetics or mechanisms of rejection that may be important in the future both in further optimizing the protection conferred by the capsule and/or developing methods for assessing capsule integrity over the device lifespan.

Overall, our findings contribute to a better understanding of the therapeutic outcomes depending on the size and composition of the follicular pool within grafted ovarian tissue. Our data show that D7 grafts—containing an abundance of primordial follicles—produce sufficient hormones to successfully restore ovarian crosstalk to both the uterus and the HPG axis while D21 grafts—containing a diminished number of immature follicles—are capable of restoring crosstalk only to the uterus and fall short of fully restoring the HPG axis. However, day 7 allografts undergo rejection before these primordial follicles have a chance to develop, suggesting that new vascular connections form and transport host T cells to the graft site at similar rates regardless of donor age. This finding is surprising since follicles are known to increase the production of pro-angiogenic cytokines [17] and some pro-inflammatory [20] cytokines as they develop. Our results thus suggest that although prepubertal ovarian tissue might be preferable to mature ovarian tissue for reconstituting lost endocrine function, their increased immunogenicity may require that rigorous immunosuppression be established at the time of transplantation.

We did not monitor hosts long enough to draw conclusions about changes in endocrine function as the graft continues to age. An important next step for the clinical translation of ovarian allografts will be methods for predicting the duration of graft function, particularly when grafts are transplanted to heterotopic sites, as factors like vascularization [21] and temperature [22] at the grafting site have been shown to influence follicle development and hormone production directly.

## 3. Materials and Methods

All procedures involving animals were performed in accordance with the protocol approved for use from 16 April 2020 by the Institutional Animal Care & Use Committee (IACUC) at the University of Michigan (00009635). Adult C57BL/6 (B6) mice (IMSR_JAX:000664) and B6CBAF1 (F1) mice (IMSR_JAX:100011) were purchased from Jackson laboratories at 8-9 weeks of age to serve as ovarian transplant recipients. 

### 3.1. Vaginal Cytology and Estrous Cycle Staging

Vaginal cytology samples were collected daily via vaginal lavage. Collection began two weeks prior to ovariectomy to confirm that all mice were cycling normally and continued until the end of the study 21 days after ovarian transplantation. Injection-grade 0.9% sodium chloride was flushed through the vaginal canal using a blunted 5 3/4 glass pipette (Fisher 13-678-20B). Samples were stored in a 48-well plate at 4 °C for up to 48 h and imaged at 10× magnification using a light microscope (Leica DMI3000 B). Estrous cycles were staged according to the proportion of leukocytes, nucleated epithelial cells and cornified epithelial cells present in samples [23]. Successful ovariectomy was confirmed prior to ovarian transplantation via observation of persistent diestrus using vaginal cytology for at least 7 days prior to transplantation. When counting number of cycles experienced after ovarian transplant, one cycle was classified as smears shifting from containing predominantly leukocytes to containing predominantly cornified cells and back again to predominantly leukocytes.

### 3.2. Blood Collection

Blood was collected via the lateral tail vein at the time points previously outlined in (Figure 1b). Mice were weighed before blood collection and the total blood volume collected from each subject did not exceed 5% of total bodyweight weekly. Mice were placed in a cylindrical restraint and the tail was swabbed 3 times with alcohol. A small incision was made perpendicular to the lateral tail vein using a #10 scalpel blade and blood was collected into 0.2 mL PCR tubes (VWR 20170-012, Atlanta GA, USA) using a sterile 5 3/4 glass pipette (Fisher 13-678-20B, Waltham MA, USA). Cardiac puncture under isoflurane anesthesia was performed to collect terminal blood. After collection, samples were left at room temperature for 90 min to minimize hemolysis during clotting. Samples were then stored at 4 °C for no more than 24 h. Serum was isolated by first running a plastic pipette tip along the interior wall of the collection tube to disrupt clot adhesion to the tube wall, followed by centrifugation at 2000× *g* for 15 min. Serum was removed from the sample surface, aliquoted and stored at −80 °C for later analysis.

### 3.3. Isolation of Ovarian Tissue for Transplantation

Ovaries for transplantation were isolated from F1 or B6 mouse pups bred in our laboratory. D7 grafts were isolated from pups 6–8 days old and D21 grafts were isolated from pups 20–22 days old. Mice were euthanized in accordance with the approved IACUC protocol (00009635). Both ovaries were removed via a midline abdominal incision using sterile surgical instruments and transferred to warm Leibovitz’s medium (Gibco 11415-064, Billings MT, USA). Syringes ½ mL in volume (BD 305620, Franklin Lakes NJ, USA) were used to carefully dissect the ovaries from the bursa. Following dissection, ovaries from 20–22-day-old mice were cut into quarters using either ½ mL syringes or a disposable #10 scalpel (Bard-Parker 372610, Franklin Lakes NJ, USA). To maintain uniformity between the cut pieces, D21 ovaries were first cut in half and each half ovary was assigned to a recipient. Slicing through the short axis of the ovary reliably produced two equally sized pieces. From there, the half ovaries were further cut into quarters prior to transplantation. In this way ovary fragments were not selected at random to be placed into each recipient. Each recipient received the tissue of ½ of a D21 ovary. Grafts were then imaged at 5× magnification using a light microscope (Leica DMI3000 B, Wetzlar, Germany) and were then immediately implanted.

### 3.4. Ovariectomy and Ovarian Transplantation

Prior to all surgical procedures, mice received 5 mg/kg carprofen as a preemptive analgesic. Mice were then anesthetized with 2% isoflurane, shaved, and prepared with three alternating washes of povidone iodine and 70% ethanol. For all recovery procedures performed under anesthesia, mice received supplemental heat from an infrared heating pad.

Host mice were 12 or 13 weeks old when ovariectomized. Briefly, a midline incision was made in the dorsal skin and the ovary was visualized through the peritoneum. A small incision was made in the peritoneum and the ovary was exposed using blunt forceps then removed via cautery with a miniature hemostat. The peritoneal incision was closed using 5-0 (Unify, #PSG-518R13, Sunnyvale CA, USA) absorbable sutures and the procedure was repeated on the opposite side for the second ovary. After removal of both ovaries, the midline incision was closed with 5-0 absorbable sutures. The closed incision was washed once more with povidone iodine. Mice received 5 mg/kg carprofen for 48 h following ovariectomy and were monitored daily for 10 days following ovariectomy. Transplantation was performed at least 14 days after ovariectomy, as soon as appropriately aged donors could be obtained from our breeding colony. All hosts were between 14 and 18 weeks old at the time of ovarian transplant.

For ovarian transplantation, a small incision was made to the left of the midline and 1–2 cm above the ovariectomy incision site. Metzenbaum scissors were used to form a small pocket in the subcutaneous space and both pieces of ovarian tissue were placed together in this pocket. The skin was closed using 5-0 absorbable sutures. Mice received 5 mg/kg carprofen for 24 h following ovariectomy and were monitored daily for 10 days.

### 3.5. Serum Hormone Quantification

Previously frozen serum samples were shipped overnight on dry ice to the University of Virginia Center for Research in Reproduction Ligand Assay and Analysis Core. Serum FSH was measured in singlicate using radioimmunoassay with a reportable range of 3–75 ng/mL and a sensitivity of 3 ng/mL. 

### 3.6. Detection of Donor-Specific Antibodies

The presence of donor-specific IgM and IgG in recipient serum was evaluated using flow cytometry. All dilutions, incubations, and washes were carried out using a solution of phosphate buffered saline containing 0.5% bovine serum albumin, 0.1% sodium azide, and 2 USP units/mL heparin sodium. Recipient serum collected at specified time points was incubated with thymocytes of donor origin, and antibodies that bound to thymocytes were detected using flow cytometry.

Thymus were extracted from 5–8-week-old B6CBAF1 (F1) females and a single-cell suspension of thymocytes was obtained via mechanical dissociation and washed 3 times. Previously frozen serum collected at time points outlined in (Figure 1) was diluted to a concentration of 1:50 in CSB and incubated with thymocytes for 30 min at 4 °C with gentle agitation. Thymocytes were washed 3 times and then incubated with anti-mouse IgM (1:250 dilution, SouthernBiotech 1020-30, Birmingham AL, USA), anti-mouse IgG (1:250 dilution, SouthernBiotech 1030-15 Birmingham AL, USA), and fixable viability stain 780 (1:1000 dilution, BD 565388 Franklin Lakes NJ, USA). After once again washing in triplicate, 100,000 events inside of the thymocyte gate were recorded on a BD FACSCanto II Franklin Lakes NJ, USA and single-color controls were used for compensation. Following the exclusion of dead cells and doublets, the mean fluorescence intensity (MFI) for each channel was calculated using FlowJo_v10.8.1. The MFI for each subject was then normalized to the MFI recorded on D0.

### 3.7. Histology and Immunohistochemistry 

A total of 21 days after implantation, ovarian grafts were removed, fixed overnight in 4% PFA in PBS at 4 °C, and then washed 3 times in PBS. Fixed samples were embedded in paraffin by the Histology Core Facility at the University of Michigan School of Dentistry using a standard overnight cycle. Embedded samples were serially sectioned at a thickness of 5 µm and affixed to Superfrost Plus slides (Fisher, 2355015) for staining. Every 5th slide was stained with hematoxylin and eosin (H&E) and stained slides were imaged using brightfield microscopy (DM1000, Leica, Wetzlar, Germany).

Three slides per subject were selected for immunohistochemistry of CD4 and CD8 based on the presence of ovarian grafts in neighboring H&E stained slides. Slides were deparaffinized and dehydrated in decreasing concentrations of ethanol before undergoing heat-mediated antigen retrieval in Tris-EDTA Buffer (Abcam, #ab94681). Slides were then washed three times in a solution of Tris-buffered saline and 0.1% tween twenty (TBS-T) and blocked for 30 min at room temperature with KPL Universal Block (Sera Care, #5560-0009, Milford MA, USA). Following blocking, slides were washed with TBS-T then incubated with either anti-mouse CD4 (Abcam, #ab183685, dilution 1:1000) or anti-mouse CD8 (Abcam, #ab203035, dilution 1:5000) diluted in Lab Vision Antibody Diluent OP Quanto (Thermo Scientific, #TA-125-ADQ, Waltham MA, USA) for one hour at room temperature. Slides were washed twice more with TBS-T and then incubated with anti-Rabbit Ig, Human ads-HRP (Southern Biotech, #4010-05 Birmingham AL, USA) diluted in Lab Vision Antibody Diluent OP Quanto (15 min at a at a 1:100 dilution for detection of CD4 or for 30 min at a 1:50 dilution for detection of CD8). Slides were again washed and then treated with DAB (BioCare Medical, #BDB2004L, Concord CA, USA) for 10 min at room temperature. Next, slides were washed with DI water, counterstained with hematoxylin (Fisher Scientific, #220-102, Waltham MA, USA) for 15 s, and then dehydrated in a graded ethanol series, beginning with deionized water, and ending with 100% ethanol. Finally, slides were clarified with xylenes and coverslipped using Permount Mounting Media (Fisher Scientific, #SP15-100).

### 3.8. Calculation of T Cell Density

To calculate T cell density at the graft site, whole slides were scanned and DAB-positive cells were calculated using ImageJ following procedures outlined in a previously published report [19]. Digital whole-slide images of IHC-stained slides were obtained at 20× magnification (scanning resolution 0.5 μm/pixel) using brightfield microscopy (Aperio AT2, Leica, Germany) and one Tiff. file for each section was manually generated without compression, using the “extract a region” function in ImageScopex64. Cell counting and density calculations were then performed using Fiji [24]. Briefly, the graft area was manually outlined, any areas containing artifacts (folding, dust, or blurring) were removed and the area remaining was calculated. Next, color deconvolution was performed using the colordeconvolution2 function [25], and example values obtained from control hematoxylin-only or DAB-only slides. Following deconvolution, the DAB channel underwent thresholding and watershedding prior to obtaining cell counts using the “analyze particle” function. 

To optimize and validate our protocol, counts and particle sizes were calculated manually for one section per mouse and compared to the results produced in Fiji. Several values were then tested for threshold, minimum, and maximum particle sizes, and the values that yielded the best agreement with the manual counts were used to analyze the remaining sections. Cell density was calculated by dividing the resulting cell count by the graft area obtained previously. An average density for each slide was then calculated, yielding 3 values per subject for CD4 and CD8. Three slides per mouse were analyzed.

### 3.9. Statistical Analysis 

Statistical analysis was performed using GraphPad Prism 9. Time to first estrus was plotted as median ± IQR and statistical significance was determined using a Kaplan–Meyer survival analysis with Bonferroni correction for multiple comparisons *p* < 0.05. The remaining data were plotted as mean ± SD and statistical significance was determined using the non-parametric Kruskal–Wallace test (*p* < 0.05) due to deviation from normal and lognormal distributions and small sample sizes. Dunn’s correction for multiple comparisons was applied when more than two groups were compared. For statistical analysis of serum FSH, samples containing FSH concentrations below the minimum value for detection were treated as the lower detection limit of the assay.

## Data Availability

The data are contained within the article and Supplementary Material.

## Supplementary Materials

The following supporting information can be downloaded at: https://www.mdpi.com/article/10.3390/ijms25063431/s1.
