# Supplementary material for: Effect of Donor Age on Endocrine Function of and Immune Response to Ovarian Grafts
Source: Int J Mol Sci. 2024 Mar 19;25(6):3431. doi: 10.3390/ijms25063431 (PMC10970747; doi:10.3390/ijms25063431)
Supplement: Supplementary file 1 [file ijms-25-03431-s001.zip › ijms-2884781-supplementary.pdf]

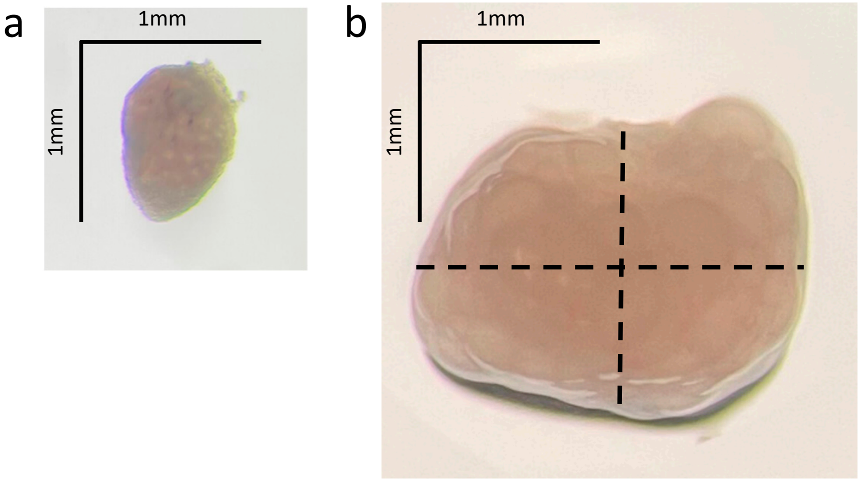

Figure S1. Ovarian tissue was isolated from 6-8 (D7) **(a)** or 20-22 (D21) **(b)** days old mice. To standardize the volume of tissue transplanted across all groups D21 ovaries were cut into quarters (dotted lines).

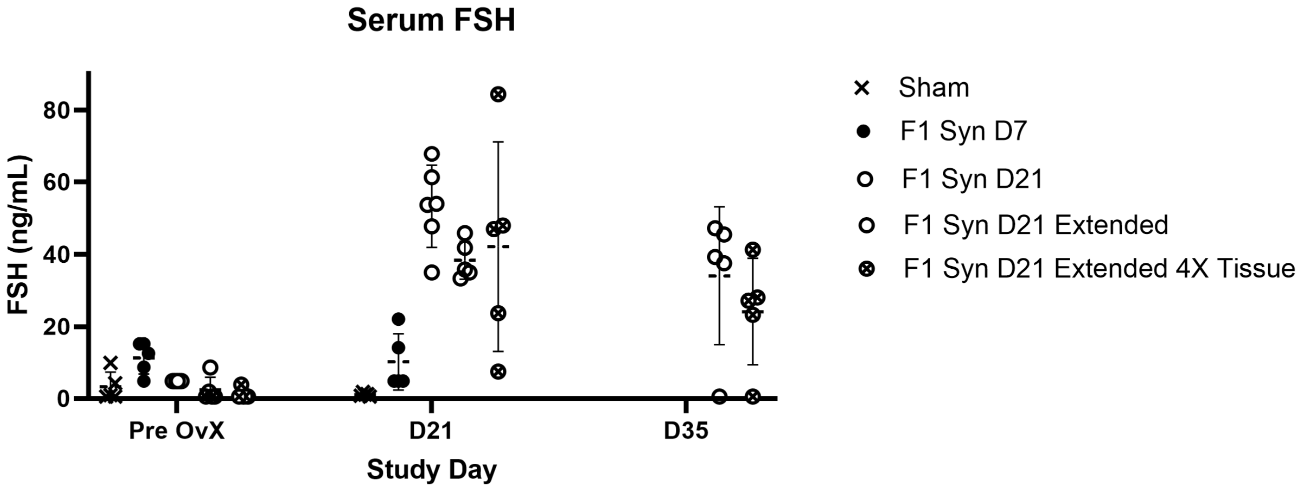

Figure S2. Only tissue from syngeneic prepubertal D7 donors was capable of restoring negative feedback to the HPG axis and suppressing FSH secretion
